# Marked Regression of Left Ventricular Hypertrophy after Outflow Desobliteration in HOCM

**DOI:** 10.1155/2012/546942

**Published:** 2012-10-02

**Authors:** Zisis Dimitriadis, Frank van Buuren, Nikola Bogunovic, Dieter Horstkotte, Lothar Faber

**Affiliations:** Department of Cardiology, Heart and Diabetes Center North Rhine-Westphalia, Ruhr University of Bochum, Georgstraße 11, 32545 Bad Oeynhausen, Germany

## Abstract

We present an HOCM patient in whom marked regression of left ventricular hypertrophy occurred within two years following outflow desobliteration by percutaneous septal ablation. Maximum wall thickness (initially documented by both echo and MRI) decreased from 34 mm to 22 mm (followup by echo only due to presence of the ICD), crossing the threshold value of 30 mm which was one of the risk markers that had triggered the primary prophylactic ICD implantation in this case prior to septal ablation.

## 1. Case Presentation

In August 2007, a physically active 34-year-old male patient presented for the evaluation of his hypertrophic obstructive cardiomyopathy (HOCM) diagnosed the year before. Exertional dyspnea (NYHA class III) correlated to a reduced oxygen uptake (VO2 peak) of 26.6 mL/kg/min (65% of the predicted value). On imaging (echocardiography and cardiac magnetic resonance imaging (MRI)), the left ventricle (LV) appeared markedly hypertrophic (maximum wall thickness: 34 mm in the anteroseptum, posterior wall: 19 mm). Gadolinium-contrast-enhanced MRI additionally revealed areas of inhomogeneous hyperenhancement in the middle and basal anteroseptal as well as the anterior walls. An outflow gradient of 70 mm Hg was measured at rest by continuous-wave Doppler echocardiography, accompanied by a septal anterior movement of the mitral valve (SAM) phenomenon with complete septal apposition and grade 1 mitral regurgitation. With a Valsalva maneuver, the outflow gradient rose to 125 mm Hg. An 48-hour Holter ECG documented a nonsustained VT. The cardiac medication included Atenolol 25 mg twice daily.

Due to presence of two risk factors [1], for sudden cardiac death, a two-chamber ICD was implanted, including the possibility to address outflow obstruction by AV sequential stimulation. With pacing the resting gradient dropped to 40 mm Hg. However, with Valsalva, the gradient still was 140 mm Hg, and symptoms did not improve. Therefore, in April 2008, percutaneous septal ablation was performed. After appropriate contrast-echo designation of the ablation region [2], 3 mL alcohol (1 mL per cm wall thickness in the target area) were injected. The postprocedural course was uneventful, with a CK peak of 785 U/L. 

**Figure 1:**
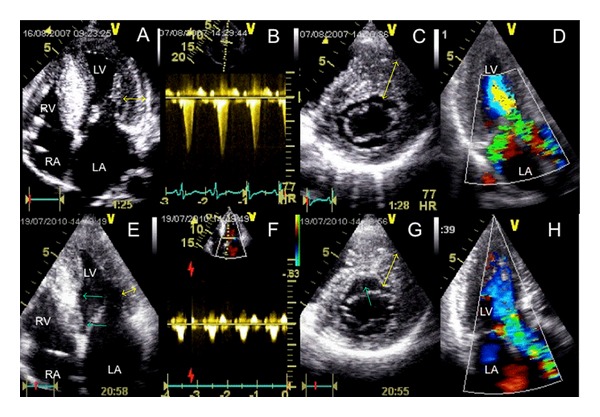
Upper panel (A–D): HOCM with severe hypertrophy of the left ventricle at baseline, particularly involving the septum ((A) and (C)) and exceeding the “critical” threshold of 30 mm (double arrow in (C)). (A) four-chamber view, (B) cw-Doppler profile of LVOT flow with a resting gradient of 70 mm Hg, (C) short-axis view in diastole with double arrow pointing to anterior septal hypertrophy, (D) apical long axis with the turbulent LVOT jet and the obstruction-associated mitral regurgitation. Lower panel (E–H): two years after successful septal ablation which has left a myectomy-like trough in the basal septum (arrows in (E) and (G)) and completely eliminated outflow gradient (F) and mitral regurgitation (H), regression of left ventricular hypertrophy can be observed both in the septum (double arrows in (G)), and the lateral wall (double arrow in (E)). LA: left atrium RA: right atrium RV: right ventricle LV left ventricle.

In July 2010, the patient reported an unrestricted exercise tolerance, correlating to a VO2max of 36 mL/kg/min (92% of the target value). On echocardiographly (Figure 1), SAM and mitral regurgitation were absent, and the outflow gradient was markedly reduced to 10 mmHg without a significant increase after Valsalva (15 mmHg). Maximum wall thickness on echocardiography was now 22 mm (anteroseptum), posterior wall thickness was 12 mm. The ICD memory revealed 9 episodes with nonsustained ventricular tachycardia events under continued *β*-Blocker medication and was empty with respect to adequate or inadequate therapeutic events. 

Our case once again demonstrates the clinical efficacy of septal ablation. In addition, it shows that the primary hypertrophic process of HOCM has an afterload-dependent component [3], in this case with a maximum posterior wall thickness going from 19 to 12 mm. Whether the decrease of the maximum wall thickness (from 34 to 22 mm) not exactly within but close to the ablation region means a modification of this patients' risk profile remains uncertain. A careful followup of all patients treated with percutaneous septal ablation with repeated risk profiling is therefore of great importance.
